# Health-Related Quality of Life and Psychological Features in Post-Stroke Patients with Chronic Pain: A Cross-Sectional Study in the Neuro-Rehabilitation Context of Care

**DOI:** 10.3390/ijerph18063089

**Published:** 2021-03-17

**Authors:** Marialuisa Gandolfi, Valeria Donisi, Simone Battista, Alessandro Picelli, Nicola Valè, Lidia Del Piccolo, Nicola Smania

**Affiliations:** 1 Neuromotor and Cognitive Rehabilitation Research Centre (CRRNC), Department of Neurosciences, Biomedicine and Movement Sciences, University of Verona, 37134 Verona, Italy; alessandro.picelli@univr.it (A.P.); nicola.vale@univr.it (N.V.); nicola.smania@univr.it (N.S.); 2 UOC Neurorehabilitation, AOUI Verona, 37134 Verona, Italy; 3 Section of Clinical Psychology, Department of Neurosciences, Biomedicine and Movement Sciences, University of Verona, 37134 Verona, Italy; valeria.donisi@univr.it (V.D.); lidia.delpiccolo@univr.it (L.D.P.); 4 Department of Neurosciences, Rehabilitation, Ophthalmology, Genetics, Maternal and Child Health, Campus of Savona, University of Genova, 17100 Savona, Italy; simone.battista@edu.unige.it

**Keywords:** psychological distress, coping strategies, health-related quality of life, chronic pain, post-stroke

## Abstract

This study aims at exploring disability, health-related quality of life (HrQoL), psychological distress, and psychological features in post-stroke patients with chronic pain. An observational cross-sectional study involving 50 post-stroke patients (25 with chronic pain and 25 without pain) was conducted. The primary outcome was the self-reported level of disability and HrQoL which were both assessed through the Stroke Impact Scale 3.0. Both psychological distress and specific psychological features (i.e., self-efficacy, coping strategies, psychological flexibility, perceived social support) were examined. Post-stroke patients with chronic pain reported statistically significant higher levels of disability and worse HrQoL, higher psychological distress and inflexibility, as well as a lower level of self-efficacy and problem-oriented coping strategies than patients without pain (*p* < 0.001). Finally, correlation analysis in the group of stroke survivors with pain showed that higher levels of disability were significantly related to higher psychological distress. This study confirms the negative influence of chronic pain on disability and HrQoL in post-stroke patients and presents preliminary insights on the association between chronic pain, disability, HrQoL, psychosocial distress, and the patient’s approach in dealing with personal difficulties and emotions. These findings carry further implications for multidisciplinary management of post-stroke patients with chronic pain.

## 1. Introduction

Pain is a common and highly disabling complaint in stroke survivors [1], more frequently so in the chronic phase than in the acute one [2]. Several types of pain are present in about 19–74% of stroke patients with a mean prevalence of 29.6% [2]. These include central post-stroke pain (CSPS) hemiplegic shoulder pain (HSP), complex regional pain syndrome (CRPS), pain related to spasticity, musculoskeletal pain and headache [3,4].

According to the International Association for the Study of Pain (IASP), pain has been defined as “[a]n unpleasant sensory and emotional experience associated with or resembling that associated with, actual or potential tissue damage” [5]. In line with this definition, the pain paradigm shifted from a biomedical one, which merely considered pain as an organic response to tissue damage, onto a biopsychosocial one, which takes into account not only the response mentioned above but which also considers pain as a complex interaction of biological, psychological, and social factors [6]. In particular, psychological distress and pain have shown a bidirectional influence of similar magnitude [7]. Psychological distress is associated with the maintenance and exacerbation of pain, mostly in chronic conditions [8]. It seems to affect patient prognosis by interfering with the adherence to the rehabilitation process [9] and the recovery from injuries [10,11], influencing the outcome of neurorehabilitation [12].

Patients who experience pain after stroke seem to be more inclined to lower levels of health-related quality of life (HrQoL) [13], worse cognitive and functional performance [14], higher fatigue perception [15], post-stroke depression, anxiety symptoms [16], and suicidality [17]. Despite its severe burden, pain is often under-diagnosed and under-treated in post-stroke survivors [18], and its clinical consequences are still inadequately understood. This may be due to the difficulties patients with aphasia, neglect syndrome, or dementia have when describing their pain experience [19,20], or to the clinicians’ abilities to analyse pain and treat it [14].

Post-stroke HrQoL depends on a comprehensive view of subjective health, including measures of the perceived physical, mental, and social well-being and functioning. Demographic factors, comorbidities, stroke severity, disability, and psychosocial factors (e.g., post-stroke depression and social support) are significant predictors of HRQoL in stroke survivors [21]. The psychological determinants of post-stroke HrQoL have only marginally been dealt with in the literature [22]. The systematic review by van Mierlo et al. [22] reported the importance of assessing psychological factors in post-stroke patients, showing that appropriate coping strategies, internal locus of control, high levels of self-efficacy, hope, and optimism were moderately associated positively with HrQoL. In contrast, negative personality characteristics (i.e., problems of temperament, problems of personality functions, and neuroticism) were moderately associated with HrQoL negatively [22]. Finally, pain is associated with poorer HrQoL, self-perceived health status, and post-stroke recovery. Up to now, the literature has only partially explored the relationship between pain, psychological distress and features such as coping strategies [23], self-efficacy [24], psychological flexibility [25] and perceived social support [26] that, in turn, can affect the HrQoL and disability [8] in stroke survivors.

In line with the statements above, this study aims (I) at determining the difference in self-reported disability and HrQoL scores, psychological distress, and psychological features between a cohort of post-stroke patients with chronic pain and a cohort of post-stroke patients without pain, and (II) at determining the association between disability and HrQoL, pain (i.e., intensity, duration, interference with life domains) and psychological distress and features in the two cohorts of post-stroke patients: with and without pain. Hence, this study will provide preliminary insights on a poorly understood field such as chronic pain experience in post-stroke patients to support the need for a multidisciplinary assessment and management of these patients in the neurorehabilitation setting.

## 2. Materials and Methods

### 2.1. Study Design and Setting

This cross-sectional study is part of the “EXPLORE” (Exploring psychological needs of patients with chronic pain attending neurorehabilitation services) project, which arose from the collaboration between the Neurorehabilitation Unit and the Clinical Psychology Unit of the Verona University Hospital (Verona, Italy). The EXPLORE project aims at investigating the psychological distress, the psychological features, and HrQoL of patients presenting different chronic pain conditions [27,28].

This study was performed according to the latest version of the Declaration of Helsinki and approved by the Ethics Committee of the Verona University Hospital, Verona (approval date: 17 January 2018, 1630CESC, Prog. No. 14112). The Strengthening the Reporting of Observational Studies in Epidemiology (STROBE) recommendations for reporting observational studies were followed [29].

### 2.2. Participants

This study included patients with chronic pain compared with a group of post-stroke patients without pain. Post-stroke outpatients were recruited according to the following selection criteria.

Inclusion criteria were age ≥18 and ≤85 years; diagnosis of stroke confirmed by a specialist in neurology and by radiologic findings (TC or RM); time from stroke ≥ three months; signature of the informed consent. Exclusion criteria were the presence of severe cognitive or communication deficits interfering with patients’ capacity to provide the informed consent or proper answers to the questionnaires. Patients with language difficulties were known to the Neurorehabilitation Unit, since they had already undergone previous speech and language rehabilitation in which their language abilities were acknowledged. After discussion with the speech and language therapists, the patients’ eligibility was further verified. In case of any unexpected communication difficulties occurring during the face-to-face assessment, the patient was excluded from the study. Patients with other neurological, orthopaedics, or medical comorbidities that could cause pain (i.e., rheumatologic disorders) and substance abusers were excluded. The patients were considered in the sub-group of stroke survivors with chronic pain if they (a) presented pain for at least three months, (b) rated their pain intensity as at least four on a 11-point Numeric Rating Scale (NRS) (0 = no pain at all; 10 = the worst pain imaginable). A score of 4 on the intensity scale identifies moderate pain, which is considered to interfere with daily living activities significantly [30]. Patients were both recruited from the Neurorehabilitation Unit of the Azienda Ospedaliera Universitaria Integrata (AOUI), Verona, Italy.

Medical charts of patient attending the Neurorehabilitation Unit between December 2018 and August 2019 were retrospectively reviewed. A physician performed an initial telephone screening interview lasting approximately 10 min that consisted of ad hoc questions to assess the presence of inclusion and exclusion criteria. During the telephone interview, patients were informed about the EXPLORE project. Moreover, the presence of pain and duration of pain in the previous three months were ascertained. Patients who fulfilled selection criteria and accepted to participate in the study were referred to a face-to-face visit at the Neurorehabilitation Unit. During this visit, they filled out a battery of paper–pencil questionnaires that investigated their sociodemographic and clinical characteristics, level of HrQoL, level of disability, pain, and psychological features.

All patients gave their written informed consent to participate in the study.

### 2.3. Variables

#### 2.3.1. Sociodemographic and Clinical Characteristics

For each patient, sex, age, educational level, civil status, and work condition were collected to describe the sample’s sociodemographic characteristics. An ad hoc clinical record was created aimed at acquiring diagnosis information, stroke onset, interest in starting a psychological intervention, as well as the duration of the multidisciplinary rehabilitative care. Moreover, a multimodal assessment was conducted aimed at analysing their HrQoL, pain perception, psychological distress, and psychological features. The assessments lasted approximately one hour. The patients were helped in the event of difficulties by the researcher. The time since stroke (in months) was collected for all patients, whereas the duration of pain (in months) was collected only in the group with pain.

#### 2.3.2. HrQoL and Disability Assessment

The self-reported level of disability and HrQoL were evaluated through the Stroke Impact Scale (SIS) 3.0 [31,32]. The SIS is a 59-item measure that investigates eight daily life activities across 8 domains: strengths, hand function, activities of daily living/instrumental activities of daily living (ADL/IADL), mobility, communication, emotion, memory and thinking, participations/role function. In this scale, patients have to rate their level of difficulty with the items, in the past 2 weeks, using a 5-point Likert Scale (1= could not to do it at all; 5= not difficult at all). Minimally clinically important differences (MCID) for strength, ADL/IADL, mobility, and hand function were 9.2, 5.9, 4.5, and 17.8, respectively [33].

#### 2.3.3. Pain Assessment

The multidimensional level of pain was evaluated through the Brief Pain Inventory (BPI) scale [34]. BPI provides the patient-reported severity of pain and the degree to which it interferes with feelings and functioning through 7 items: activity in general, mood, ability to walk, ability to work, relationships with other people, sleep, and the taste for life. Each item is rated using a numerical rating scale from 0 (“does not interfere”) to 10 (“completely interferes”). A body chart is used to localise pain.

#### 2.3.4. Psychological Assessment

The psychological assessment was performed in line with the EXPLORE protocol [27,28]. Psychological distress was measured through the Symptom Checklist-90 (SCL-90-R) scale measuring psychopathological symptoms through 90 items rated on a five-point Likert scale (from “not at all” to “extremely”) [35,36]. The global severity index (GSI), which reflects the overall measure of psychological distress, was calculated from this scale (higher scores = higher psychological distress).

The level of self-efficacy was evaluated through the General Self-Efficacy (GSE) scale, which is a 10-item scale rating “people’s optimistic self-beliefs to cope with a variety of difficult demands in life” on a four-point Likert-type scale (higher scores = higher levels of perceived self-efficacy) [37]. Coping strategies were assessed through the Coping Orientation to Problems Experienced (COPE), a 60-item scale rated on a four-point scale (from “usually I do not do this at all” to “usually I do this a lot”), which analysed positive attitude, social support, problem-solving, avoidance strategies, and turning to religion coping strategies (higher scores = higher use of those coping strategy) [38]. Psychological inflexibility, which is the “the phenomenon that occurs when a person is unwilling to remain in contact with particular private experiences (e.g., bodily sensations, emotions, thoughts, memories, images, behavioural predispositions) and takes steps to alter the form or frequency of these experiences or the contexts that occasion them, even when these forms of avoidance cause behavioral harm” [39] was explored through the Acceptance and Action Questionnaire II (AAQ-II), which measures this construct through a 7-item scale based on a seven-point Likert scale from “never true” to “always true” (higher scores = higher levels of psychological inflexibility) [40]. Finally, the perceived social support was measured through the Multidimensional Scale of the Perceived Social Support (MSPSS), which investigates the perceived social support by family, friendships, and significant others through a 12-item scale based on a seven-point Likert scale (higher scores = higher levels of social support perceived) [41].

### 2.4. Bias

Potential sources of bias were addressed. Firstly, during the medical chart revision, the presence of post-stroke pain was not always reported. Hence, patients attending the Neurorehabilitation Unit in the reference period were contacted by phone for further investigations. Secondly, to avoid any specific influences in reporting data of the patients who decided to participate in the study, the telephone interviews were performed by two physicians who had never been in contact with them before. Moreover, an ad hoc telephone questionnaire was set up to be consistent among patients on the information collection. We cannot exclude a selection and questionnaire administration bias. The patients who were recruited and accepted to participate in the study should be not representative of all stroke survivors’ patients since, for example, they reported a level of cognitive functioning adequate to fill out questionnaires. Moreover, we cannot exclude that they were already more willing toward a possible psychological intervention and to undertake the psychological assessment. The fixed order of providing the questionnaire might be acknowledged as a possible administration bias. All the questionnaires used were validated instruments for the Italian context and already used in the context of neurological diseases [27,28,42,43]. The fixed administration order might have interfered in the results.

### 2.5. Statistical Methods

Descriptive statistics included frequency tables, means, and standard deviation (SD). Parametric or non-parametric tests were used for inferential statistics according to the data distribution (Shapiro–Wilk test). Outliers, defined as values laying two standard deviations above the mean values of the group, were excluded. The *t*-test for independent samples (or the Mann–Whitney test) was used to test statistical differences in demographic and clinical outcomes between the two groups. The Fisher test was used to check differences in sex between the two groups. A Pearson’s correlation (or Spearman’s correlation) was run to determine the relationship between the severity of disability and HRQoL assessed by the SIS, the BPI, and psychological features. The correlation strength was defined as very high (ρ > 0.9), high (ρ = 0.7–0.89), moderate (ρ = 0.5–0.69), low (ρ = 0.3–0.49), or very low (ρ < 0.29) [44]. The *p*-value for significance was set at 0.05. Bonferroni correction was applied for multiple correlation analyses on the same dependent variable. When exploring the association between the SIS (total score or each domain) and pain, the alpha level for significance was set at 0.00625. When exploring the association between the SIS (total score or each domain) and psychological outcomes, the alpha level was set at 0.004545. Statistics analyses were carried out through SPSS 26.0 (IBM SPSS Statistics, Version 22.0, 2013, Armonk, NY, USA).

## 3. Results

### 3.1. Participants

A total of 247 in-hospital clinical records were reviewed. Fifty-four patients were excluded because they did not meet the inclusion criteria. In the remaining 193 medical charts, 77 patients reported the presence of pain, of which 36 reported a pain intensity ≥4 (NRS) for at least three months. Among this group, 11 patients refused to participate in the study, so 25 patients with chronic post-stroke pain were included. In line with that, patients without chronic post-stroke pain were randomly contacted until reaching a sample of 25 patients per group. The randomisation process was done by assigning to the 116 medical charts of patients without pain a serial number from 1 to 116, from which 25 patients were extracted randomly through the ‘Random’ Excel function.

### 3.2. Sociodemographic and Clinical Characteristics

The final sample was composed by 50 post-stroke outpatients (age range: 47–83; years; mean 63.96 ± 9.59 SD), of those 25 with chronic pain (age range: 47–81; years; mean 62.8 ± 9.18 SD) and 25 without pain (age range: 48–83; years; mean 65.1 ± 10.04 SD). Demographic and clinical characteristics are reported in Table 1.

All patients were assisted by family members and were living in their home. No patients were institutionalised or living in a nursing home. In this cohort, 32% were female, and 20% were employees. Most patients were married (76%) with high-school qualification (34%). As far as the site of lesion was concerned, 48% were left lesion, 48% were right lesion, and 2% were bilateral. Most patients suffered from Partial Anterior Circulation Syndrome (61%) due to ischemic lesion (75%). Aphasia and neglect were present in 24% and 14% of patients, respectively. Patients had been on a rehabilitation program for neurorehabilitation on average for 26.43 months. More than half of the sample (66%) was interested in receiving psychological support.

Patients with chronic pain did not differ for age and sex from patients without pain. Despite this, they had a significantly lower education level (*p* = 0.002) as well as a higher percentage of ischemic stroke (*p* = 0.023) and aphasia (*p* = 0.04) than patients without pain. No significant between-group differences were found regarding the employment condition, civil status, brain lesion side and site, duration of the rehabilitation program, and interest in receiving psychological support.

In the group with pain, the intensity was moderate-to-severe with an NRS mean intensity score of 6.4 (SD: 1.60) and a mean duration of 21.48 (SD: 29.71) months. The most frequent type of pain was musculoskeletal pain, affecting six patients (24%), followed by shoulder pain, central post-stroke pain, and headache syndromes in five patients (20%), spasticity-related pain in three patients (12%), and complex regional pain syndrome in one (4%) patient. The mean time with pain was 21.48 (SD: 29.7) months. Pain occurred on average 15.04 (SD: 23.89) months after stroke onset. The results of BPI are reported in Table 2.

### 3.3. HrQoL and Disability

The SIS scores gathered from the overall sample as well as in the two cohorts of analysed patients are reported in Table 3.

Patients with chronic pain reported significantly lower score (higher disability) in the SIS total score (*p* < 0.001), memory and thinking (*p* < 0.001), emotion (*p* = 0.024), communication (*p* < 0.01), ADL/IADL (*p* = 0.008), hand function (*p* = 0.016), and participation/role function (*p* < 0.001) compared to patient without pain (Table 3).

### 3.4. Psychological Distress and Psychological Features

Psychological distress and psychological features scores are reported in Table 3. Patients with chronic pain reported significantly higher GSI scores (*p* = 0.001) compared to patients without pain, indicating a higher level of psychological distress. In particular, 10 (40%) stroke survivors with chronic pain and three (12%) without pain obtained a GSI score > 0.57, which is considered a cut-off for the presence of psychological distress [28,45,46]. The group with chronic pain reported lower GSE (*p* = 0.015), lower COPE-Problem Oriented (*p* < 0.001), and higher AAQ-II (*p* = 0.005) scores than the patient without pain.

### 3.5. Association between Pain, Disability, HrQoL, and Psychological Features

In patients with chronic pain, the pain intensity assessed by the BPI and pain duration were moderate and strongly correlated with the length of care respectively (r = 0.75, *p* < 0.001; r = 0.46, *p* = 0.019). Moreover, a low negative correlation between length of care and the strength domain (r = −0.34, *p* = 0.017) was reported. Correlations between SIS and BPI are reported in Table 4. Regarding the pain intensity, a negative correlation was found between the NRS and the SIS total (r = −0.4; *p* = 0.001) and the single domain scores concerning participation/role function (r = −0.502; *p* < 0.001) and memory and thinking (r = −0.408; *p* = 0.003). Negative correlation was found between the SIS total score and the BPI for interference with mood (r = −0.587; *p* = 0.002).

Table 5 shows the correlations between the Stroke Impact Scale and psychological features in the group with pain. There was a moderate to strong significant association between the SIS total score and GSI (r = −0.64; *p* = 0.001). Specifically, the SIS sub-scores referring to emotions (r = −0.64; *p* = 0.001) and participation (r = −0.63; *p* < 0.001) showed a negative correlation with the GSI. Regarding coping strategies, the SIS sub-scores referring to memory and thinking were significantly correlated with the COPE-PO (r = 0.55; *p* < 0.001). In the framework of psychological inflexibility, in the group with pain, there was a significant negative correlation between the SIS sub-scores in the emotion domains (r = −0.65; *p* < 0.001), and participation (r = −0.53; *p* < 0.001) and the AAQ-II.

## 4. Discussion

In this explorative study, we found evidence to support the negative influence of chronic pain on the self-reported levels of disability and HrQoL in post-stroke patients with and without pain. Our findings provide preliminary evidence to support an association between psychosocial features, the level of disability, the level of HrQoL, and chronic pain in post-stroke patients.

By analysing the differences between the cohort with pain and the cohort without pain, the former reported higher levels of disability in the SIS total score and in the different domains. The differences in ADL/IADL, mobility, and hand function reached the MCID (16.8, 7.56, and 21.28, respectively), indicating that disability severity in patients with chronic pain was significantly higher, also from a clinical point of view [32]. A higher disability in the cognitive domain may depend on the inclusion in the study of patients with aphasia.

The fact that patients with chronic pain did not differ from patients without pain in muscle strength and mobility performance suggests that the self-reported perception of general motor aspects were apparently not affected by chronic pain. In contrast, a self-reported higher disability was reported on hand function in chronic pain patients, confirming that upper limb pain syndromes mostly affect post-stroke patients with a negative impact on disability [4]. However, despite the equal perceived general motor disability, patients with chronic pain reported higher disability levels in memory and emotional domains. This was also highlighted by the fact that the patients with pain reported higher psychological distress, as measured by the SCL-90, than the patients without pain, with a severity that was significantly correlated to higher self-reported disability in the overall SIS score, and some single domains.

As regards psychological distress, a previous study by Zhang et al. showed that the mean level obtained at the SCL-90 was higher in post-stroke patients compared to the control group [43]. In our study, using the same instrument, we have also noticed how post-stroke patients with chronic pain reached even higher levels of psychological distress compared to the ones without pain. The result is coherent with many studies highlighting that pain increases the burden related to psychological distress [7,47]. However, research has shown that the relationship between psychological distress and pain is bidirectional: the latter is a positive predictor of the former, and the former increases the risk of developing the latter and influences its perception. In the presence of chronic pain, psychological distress resulted significantly associated with SIS total score and participation and emotions domains. These results suggest the importance of intervening in the assessment and in the reduction of psychological distress through a multidisciplinary approach when dealing with patients with pain.

Considering the other psychological features, chronic pain in post-stroke patients was associated with lower levels of self-efficacy, psychological flexibility, and problem-oriented coping strategies, therefore suggesting a more dysfunctional approach in dealing with personal difficulties, stress, and emotions. Self-efficacy beliefs for people experiencing chronic pain can negatively modify their expectation about their ability to perform a particular activity and their confidence to accomplish that task despite the pain. According to literature, lower self-efficacy levels are consistently related to greater clinical pain ratings in various chronic pain conditions [8]. However, this is the first study that investigated the relation between self-efficacy with chronic pain in post-stroke patients. As far as self-efficacy in post-stroke survivors is concerned, recent literature suggests positive results in the introduction of motivational interview communication approaches to support patients’ self-efficacy in adjusting to stroke consequences and in identifying realistic personal goals in the recovery process [48]. Furthermore, a lower level of psychological flexibility was found within the subgroup of post-stroke patients with chronic pain. Psychological flexibility is defined as “the ability to contact the present moment more fully as a conscious human being, and to change or persist in behaviour when doing to serve valued ends” [49]. These results are in line with recent literature in the context of chronic pain management. High levels of psychological flexibility seem to reduce the impact of chronic pain, so that therapy that addresses such psychological process, such as the Acceptance and Commitment Therapy (ACT), may enhance the patients’ care pathway [50]. Moreover, also when dealing with stroke, acceptance of the stroke condition and its consequences presented a significant role in the process of stroke adjustment [51]. A recent preliminary experience of a brief group-based ACT study aimed at improving psychological flexibility reported promising results for stroke survivors and might be further tailored to the needs of stroke survivors with chronic pain [52]. Finally, coping strategies—the cognitive and behavioural efforts to manage problems and reduce stress [53]—have already been considered as an important psychological factor related to HrQoL in post-stroke patients [54,55,56]. In healthy participants, adaptive coping strategies have been shown to be inversely related to depression, while after stroke, coping strategies and depression scores were independently associated with the psychological health of chronic patients [57]. Moreover, post-stroke patients make lower use of active problem-oriented coping strategies than patients with other brain damage aetiologies [58], and the use of avoidance behaviour is a predictor of depression at discharge from the rehabilitation ward [59]. Our results showed that the hypothetical psychological profile of post-stroke patients with chronic pain is characterised by significantly lower problem-oriented strategies, which are associated with a tendency to higher avoidance strategies and lower positive attitude than post-stroke patients without pain. However, a previous qualitative study highlighted that stroke survivors with pain represents a non-homogenous group, and assessment of their specific coping strategies in relation to the pain they experience should be introduced in the clinical encounter [60]. Therefore, further studies should verify our results in a larger sample in order to deepen the understanding of the differences in coping styles, and thus contribute to foster specific psychological interventions.

Due to the small sample of patients, we cannot identify demographic or clinical factors associated with the presence of post-stroke pain. However, in our small sample, patients with chronic pain presented lower levels of education, a higher percentage of ischemic stroke, and a higher percentage of aphasia. A low formal education level is commonly associated with a higher prevalence of several chronic pain conditions [61] and low health literacy, which is defined as “the cognitive and social skills which determine the motivation and ability of individuals to gain access to, understand and use information in ways which promote and maintain good health” [62]. Mackey et al., in their work, highlighted that low health literacy could jeopardise the patients’ possibilities to develop self-management skills that are fundamental in the treatment of chronic diseases [63].

The higher level of post-stroke pain in patients suffering from aphasia is in line with the one reported in the literature [64]. People with aphasia after stroke are less able or completely unable to express their pain, due to language, speech, and cognitive impairment. This can also be due to the difficulty with the self-report assessment scale for pain, so it becomes important for clinicians to better investigate the presence of pain in aphasia more appropriately [64]. In line with this, we cannot exclude that the higher disability reported in the communication domain depends on the higher percentage of patients having aphasia in the group with chronic pain. Our results foster the need for a feasible, reliable, and valid instrument to assess pain, even in patients with aphasia after stroke.

Patients with chronic pain had a longer duration of multidisciplinary rehabilitation care (in our sample twice as long as patients without pain) and therefore, more significant care effort also from an economic perspective [65]. Longer pain duration and higher pain intensity resulted in a longer duration of the rehabilitation care, and it is associated with functional dependency at discharge from hospital, depression, and restricted mobility in the long term [66]. It is essential that clinicians recognise post-stroke pain earlier to implement all the strategies necessary to avoid its chronification but also to improve HrQoL.

The implication of these findings is relevant for a multidisciplinary assessment and management of chronic post-stroke patients. The fact that with the same perceived motor disability (mobility and strength), patients with chronic pain reported a greater disability in the domains related to emotional aspects emphasises the need for psychological interventions to promote a better emotional adjustment to the stroke experience and its consequences. This suggestion is confirmed also by the positive attitude of patients regarding psychological help, with the majority of the sample reporting interest for psychological interventions. Considering the psychological characteristics investigated in the current study, the psychological intervention might be targeted to promote problem-oriented coping strategies, psychological flexibility, and self-efficacy. However, further studies with a larger sample are needed to confirm our results.

### Strengths and Limitations

One of the strengths of this study is that it explored disability, HrQoL, and psychological distress and features in a cohort of patients generally overlooked in the literature and often under-recognised in clinical practice. Another strength of the study is the comprehensive protocol to explore diverse psychological aspects that can be impaired in patients with chronic pain and then addressed by multidisciplinary management. Moreover, the use of patient-reported outcome measures to evaluate the self-reported level of disability and HRQoL (SIS) should also be considered as a strength of the study. This instrument reports high validity, since an excellent correlation has been reported in post-stroke patients between the SIS cognitive factors and the MMSE, between the SIS physical factors and the Barthel Index and instrumental ADL, and between SIS emotional factor and anxiety and depression [31].

The limitations of this study are the small sample of patients, the lack of pre-registration of the study, the cross-sectional nature of the study, which did not allow for an evaluation of the causative relationship between pain, HrQoL, and psychological distress, and the lack of information about the pharmacological treatment of the patients. Moreover, the patients who were recruited and accepted to participate in the study are not representative of all stroke survivors’ patients, since, for example, they reported a level of cognitive functioning adequate to fill out questionnaires. Moreover, we cannot exclude that they were already more willing to take part in a possible psychological intervention and to undergo psychological assessment. Finally, we cannot exclude a selection and questionnaire administration bias.

## 5. Conclusions

Chronic pain is a common symptom in post-stroke patients increasing patients’ disability and affecting rehabilitation outcomes. To date, the management of these patients is a challenge in the neurorehabilitation setting [67]. As a matter of fact, it is important to deeply understand all the factors involved in pain occurrence and chronification as well as its impact on disability and HrQoL. The evaluation of specific clinical and psychological needs is unmet in post-stroke patients with chronic pain. With the limitation of a small sample size, our cross-sectional study highlighted the potential negative effect of pain on different domains of life, together with the association with higher psychological distress, low levels of problem-oriented coping strategies, self-efficacy and psychological flexibility. Within the integrated bio-psycho-social approach, psychological distress and psychological features should be assessed and managed early in post-stroke survivors to improve the rehabilitative care and outcomes.

## Figures and Tables

**Table 1:** Patients sociodemographic and clinical characteristics.

	Total Sample *n* = 50	Group with Pain *n* = 25	Group without Pain *n* = 25	Between-Group Differences *p*-Value
Age (years) (mean, SD)	63.96 (9.59)	62.8 (9.18)	65.1 (10.04)	0.398
Sex (female) (%)	16 (32%)	8 (32%)	8 (32%)	1
Employment (yes) (%)	10 (20%)	4 (16%)	6 (24%)	0.478
Qualification (%)				0.002 *
Primary School	13 (26%)	7 (28%)	6 (24%)	0.747
Secondary School	16 (32%)	13 (52%)	3 (12%)	0.002 *
High-School	17 (34%)	5 (20%)	12 (48%)	0.037 *
Academic Degree	4 (8%)	0 (0%)	4 (16%)	0.037 *
Civil status (%)				0.308
Unmarried	2 (4%)	0 (0%)	2 (8%)	0.147
Married	38 (76%)	20 (80%)	18 (72%)	0.508
Divorced/Widowed	10 (20%)	5 (20%)	5 (20%)	1.000
Brain injury side (%)				0.165
Right	24 (48%)	10 (40%)	14 (56%)	0.258
Left	24 (48%)	13 (52%)	11 (44%)	0.571
Bilateral	2 (4%)	2 (8%)	0 (0%)	0.149
Brain injury site (%)				0.88
TACS	2 (4.76%)	2 (9.52%)	0 (0%)	0.147
PACS	26 (61.9%)	15 (71.43%)	11 (52.38%)	0.204
LACS	12 (28.57)	3 (14.29%)	9 (42.86%)	0.040 *
POCS	2(4.76%)	1(4.76%)	1 (4.76%)	1.000
Stroke Type (%)				0.023 *
Ischemic	36 (75%)	21 (84%)	15 (65.2%)	0.133
Haemorrhagic	10 (20.8%)	2 (8%)	8 (34.8%)	0.022 *
Mixed	2 (4.2%)	2 (8%)	0 (0%)	0.166
Aphasia (yes) (%)	12 (24%)	10 (40%)	2 (8%)	0.042 *
Neglect (yes) (%)	7 (14%)	6 (24%)	1 (4%)	0.119
Time since stroke (months) (mean, SD)	28.54 (31.33)	36.56 (37.78)	20.52 (21.03)	0.12
Taking charge duration (months) (mean, SD)	26.43 (30.54)	33.9 (36.5)	18.3 (20.1)	0.078
Interest in psychological support (yes) (%)	33 (66%)	16 (64%)	17 (68%)	0.765

Legend: *, statistically significant; N, number; SD, standard deviation; %, percentages; TACS, Total Anterior Circulation Syndrome; PACS, Partial Anterior Circulation Syndrome; LACS, Lacunar Syndromes; POCS, Posterior Circulation Syndrome; NRS, Numeric Rating Scale.

**Table 2:** Brief Pain Inventory scores.

Section	Mean (SD) *n* = 25
Worst pain in last 24 h	6.24 (2.83)
Least pain in last 24 h	2.24 (2.31)
Pain on average in the last 24 h	4.60 (2.23)
Pain right now	3.00 (3.65)
Interference with general activity	3.83 (2.16)
Interference with mood	4.03 (2.58)
Interference with normal work (including housework)	4.25 (2.91)

Legend: N, number; SD, standard deviation; NRS, Numeric Rating Scale.

**Table 3:** Stroke Impact Scale scores; Disability and health-related quality of life.

	Total Sample *n* = 50 Mean (SD)	Pain Group *n* = 25 Mean (SD)	No Pain Group *n* = 25 Mean (SD)	Between-Group Differences *p*-Value
Stroke Impact Scale				
Total score	238.64 (41.77)	218.08 (40.16)	259.20 (35.37)	<0.001 *
Strength	51.60 (20.51)	47.60 (18.55)	55.60 (21.95)	0.17
Memory and thinking	64.97 (18.33)	56.11 (20.32)	73.83 (10.49)	<0.001 *
Emotion	56.13 (10.78)	52.71 (11.73)	59.56 (8.68)	0.024 *
Communication	67.49 (17.38)	61.26 (18.20)	73.71 (14.31)	0.01 *
ADL/IADL	74.56 (22.88)	66.16 (22.34)	82.96 (19.90)	0.008 *
Mobility	60.49 (17.67)	56.71 (15.78)	64.27 (18.93)	0.132
Hand function	43.12 (31.83)	32.48 (29.38)	53.76 (31.14)	0.016 *
Participation/role function	56.05 (20.91)	45.10 (20.65)	67.00 (15.73)	<0.001 *
Psychological distress and features				
GSI	0.42 (0.35)	0.59 (.40)	0.25 (0.19)	0.001 *
GSI ≥ 0.57	13 (26%)	10 (40%)	3 (12%)	0.050 *
GSE	32.7 (5.96)	30.68 (6.03)	34.72(5.27)	0.015 *
COPE-SS	29.72 (8.11)	28.24 (7.41)	31.20 (8.66)	0.2
COPE-AS	25.82 (6.87)	26.68 (6.12)	24.96 (7.57)	0.382
COPE-PA	34.44 (6.59)	33.44 (7.41)	35.44 (5.63)	0.288
COPE-PO	35.84 (8.77)	30.04 (6.76)	41.64 (6.43)	<0.001 *
COPE-TR	21.98 (6.20)	20.88 (4.84)	23.08 (7.25)	0.214
AAQ-II	16.54(10.61)	20.64 (11.28)	12.44 (8.22)	0.005 *
MSPSS Family	26.44 (2.67)	26.08 (2.66)	26.80 (2.69)	0.346
MSPSS Friends	19.02 (8.25)	19.32 (7.35)	18.72 (9.20)	0.8
MSPSS Other	24.82 (4.83)	25.6 (3.11)	24.04 (6.06)	0.26

Legend: *, statistically significant; N, number; SD, standard deviation; ADL, activity of daily living; IADL, instrumental activity of daily living; %, percentage; GSI, Global Severity Index; GSE, General Self-Efficacy; COPE, coping style; SS, social support; AS, avoidance strategies; PA, positive attitude; PO, problem oriented; TR, turning to religion; AAQ-II, Acceptance and Action Questionnaire II; MSPSS, Multidimensional Scale of Perceived Social Support.

**Table 4:** Association between the Stroke Impact Scale scores and pain outcomes (*n* = 25).

Stroke Impact Scale									
	Total Score	Strength	Memory and Thinking	Emotion	Communication	ADL/IADL	Mobility	Hand Function	Participation/Role Function
NRS	−0.4 *	−0.13	−0.41 *	−0.3	−0.30	−0.36	−0.22	−0.38	−0.50 *
Least pain	−0.01	0.04	−0.20	−0.16	−0.01	0.05	0.11	0.17	0.06
Average pain	−0.4	−0.1	−0.11	−0.16	−0.13	0.15	0.08	0.06	0.06
Pain right now	0.04	0.16	−0.25	−0.18	0.01	0.08	0.11	0.26	0.02
Worst pain	0.10	0.09	−0.14	−0.16	−0.06	0.32	0.28	0.23	0.15
General activity	−0.30	0.02	−0.20	−0.25	−0.90	0.12	0.14	0.15	0.08
Mood	−0.59 *	−0.18	−0.52	−0.48	−0.40	−0.43	−0.5	−0.02	−0.51
Normal work	−0.46	−0.18	−0.24	−0.36	−0.16	−0.31	−0.42	−0.29	−0.44

Legend: * Correlation is significant at *p* ≤ 0.00625 (2-tailed); ADL, activity daily living; IADL, instrumental activity daily living.

**Table 5:** Association between the Stroke Impact Scale scores and psychological outcomes (*n* = 25).

	Stroke Impact Scale								
	Total Score	Strength	Memory and Thinking	Emotion	Communication	ADL/IADL	Mobility	Hand Function	Participation/Role Function
GSI °	−0.64 *	−0.46	−0.51	−0.64 *	−0.33	−0.448	−0.37	−0.15	−0.63 *
GSES	0.33	0.18	0.46	0.35	0.30	0.3	0.02	−0.18	0.31
COPE-SS	−0.19	−0.41	0.11	−0.08	0.15	−0.15	−0.17	−0.33	−0.23
COPE-AS	−0.48	−0.49	−0.36	−0.34	−0.11	−0.31	−0.28	−0.36	−0.35
COPE-PA	0.26	0.01	0.18	0.32	0.29	0.22	−0.09	0.12	0.27
COPE-PO	0.179	0.01	0.55 *	0.08	0.37	0.09	0.07	−0.39	0.16
COPE-TR	0.04	0.12	0.07	−0.06	0.06	0.08	−0.23	0.18	−0.02
AAQQ-II °	−0.48	−0.33	−0.48	−0.65 *	−0.21	−0.35	−0.17	−0.08	−0.53 *
MSPSS-Family °	0.13	0.05	0.35	0.26	−0.09	0.17	0.21	−0.26	0.14
MSPSS-Friends °	−0.02	−0.22	0.04	0.17	0.08	−0.08	0.09	−0.08	−0.07
MSPSS-Others °	0.09	0.02	0.31	0.16	−0.12	0.16	0.29	−0.15	0.03

Legend: * Correlation is significant at *p* ≤ 0.004545 (two-tailed); ADL, activity of daily living; IADL, instrumental activity of daily living; %, percentage; GSI, Global Severity Index; GSE, General Self-Efficacy; COPE, coping style; SS, social support; AS, avoidance strategies; PA, positive attitude; PO, problem oriented; TR, turning to religion; AAQ-II, Acceptance and Action Questionnaire II; MSPSS, Multidimensional Scale of Perceived Social Support; °, Spearman’s correlation.

## Data Availability

The data presented in this study are available on request from the corresponding author.
